# Hypothalamic POMC or MC4R deficiency impairs counterregulatory responses to hypoglycemia in mice

**DOI:** 10.1016/j.molmet.2018.11.004

**Published:** 2018-11-20

**Authors:** Benjamin P. Tooke, Hui Yu, Jessica M. Adams, Graham L. Jones, Talisha Sutton-Kennedy, Lakshmi Mundada, Nathan R. Qi, Malcolm J. Low, Kavaljit H. Chhabra

**Affiliations:** 1 Case Western Reserve University, Cleveland, OH, USA; 2 Department of Molecular and Integrative Physiology, University of Michigan Medical School, Ann Arbor, MI, USA; 3 Department of Pediatrics, University of Michigan, Ann Arbor, MI, USA; 4 Neuroscience Graduate Program, University of Michigan, Ann Arbor, MI, USA; 5 Department of Internal Medicine, Division of Metabolism, Endocrinology and Diabetes, University of Michigan Medical School, Ann Arbor, MI, USA; 6 Department of Medicine, Division of Endocrinology, Diabetes and Metabolism, University of Rochester School of Medicine and Dentistry, Rochester, NY, USA

**Keywords:** Hypothalamus, Pro-opiomelanocortin (POMC), Melanocortin 4 receptor (MC4R), Hypoglycemia counterregulation, Diabetes

## Abstract

**Objective:**

Life-threatening hypoglycemia is a major limiting factor in the management of diabetes. While it is known that counterregulatory responses to hypoglycemia are impaired in diabetes, molecular mechanisms underlying the reduced responses remain unclear. Given the established roles of the hypothalamic proopiomelanocortin (POMC)/melanocortin 4 receptor (MC4R) circuit in regulating sympathetic nervous system (SNS) activity and the SNS in stimulating counterregulatory responses to hypoglycemia, we hypothesized that hypothalamic POMC as well as MC4R, a receptor for POMC derived melanocyte stimulating hormones, is required for normal hypoglycemia counterregulation.

**Methods:**

To test the hypothesis, we induced hypoglycemia or glucopenia in separate cohorts of mice deficient in either POMC or MC4R in the arcuate nucleus (ARC) or the paraventricular nucleus of the hypothalamus (PVH), respectively, and measured their circulating counterregulatory hormones. In addition, we performed a hyperinsulinemic-hypoglycemic clamp study to further validate the function of MC4R in hypoglycemia counterregulation. We also measured *Pomc* and *Mc4r* mRNA levels in the ARC and PVH, respectively, in the streptozotocin-induced type 1 diabetes mouse model and non-obese diabetic (NOD) mice to delineate molecular mechanisms by which diabetes deteriorates the defense systems against hypoglycemia. Finally, we treated diabetic mice with the MC4R agonist MTII, administered stereotaxically into the PVH, to determine its potential for restoring the counterregulatory response to hypoglycemia in diabetes.

**Results:**

Stimulation of epinephrine and glucagon release in response to hypoglycemia or glucopenia was diminished in both POMC- and MC4R-deficient mice, relative to their littermate controls. Similarly, the counterregulatory response was impaired in association with decreased hypothalamic *Pomc* and *Mc4r* expression in the diabetic mice, a phenotype that was not reversed by insulin treatment which normalized glycemia. In contrast, infusion of an MC4R agonist in the PVH restored the counterregulatory response in diabetic mice.

**Conclusion:**

In conclusion, hypothalamic *Pomc* as well as *Mc4r*, both of which are reduced in type 1 diabetic mice, are required for normal counterregulatory responses to hypoglycemia. Therefore, enhancing MC4R function may improve hypoglycemia counterregulation in diabetes.

## Highlights

• Hypothalamic POMC as well as MC4R is necessary to counteract hypoglycemia.
• Type 1 diabetic mice exhibit a reduced *Pomc* and *Mc4r* expression in the hypothalamus.
• Insulin treatment does not restore *Pomc* and *Mc4r* expression in diabetic mice.
• MC4R agonist improves hypoglycemia counterregulation in diabetic mice.

## Graphical abstract

**Figure:**
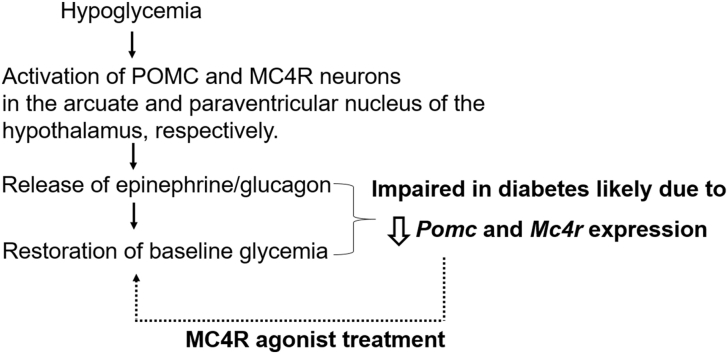

## 1 Introduction

Patients with diabetes are at high risk for life threatening hypoglycemia due to insulin therapy or other drugs that increase insulin secretion, which may lower blood glucose below 60–70 mg/dl [1]. Nearly 30–40% and 15–20% of patients with type 1 and type 2 diabetes on insulin therapy, respectively, suffer from severe hypoglycemia with an incidence of 1.0–1.7 episodes per patient per year [2], [3], [4], because the physiological counterregulatory response to hypoglycemia is impaired in diabetes. The primary physiological counterregulatory response to hypoglycemia includes rapid increases in plasma epinephrine and glucagon levels. Subsequently, blood glucose is restored to normal. These coordinated responses are blunted in patients with diabetes [5]. Altogether, impaired hypoglycemia counterregulation limits the management of diabetes.

To correct blunted counterregulatory response in diabetes, it is essential to understand the physiology underlying the response in health and diabetes. The hindbrain [6] or ventromedial nucleus of the hypothalamus (VMH) [7], [8], [9], [10], [11], [12], [13], [14] have been the focus of research pertaining to hypoglycemia counterregulation. Recently, neurons in the parabrachial nucleus were also reported to contribute to hypoglycemia counterregulation [15], [16]. However, a precise molecular pathway mediating the physiological counterregulatory response to hypoglycemia remains to be determined.

The hypothalamic melanocortin system, consisting predominantly of a proopiomelanocortin (POMC)/melanocortin 4 receptor (MC4R) circuit, is involved in the activation of the sympathetic nervous system (SNS) [17], [18], [19], [20], [21], [22], [23], [24], [25], which, in turn, is essential for hypoglycemia counterregulation [5]. Yet, the necessity of hypothalamic POMC or MC4R in hypoglycemia counterregulation is unknown. Therefore, in this study, we assessed the function of POMC and MC4R in hypoglycemia counterregulation using mice deficient in POMC in the arcuate nucleus (ARC) or MC4R in the paraventricular nucleus of the hypothalamus (PVH). Moreover, we elucidated their contribution to impaired counterregulatory responses to hypoglycemia in diabetes using streptozotocin (STZ) induced type 1 diabetes mouse model or non-obese diabetic (NOD) mice.

## 2 Methods

### 2.1 Animal care and use

All procedures were approved by the University of Michigan Institutional Committee on the Use and Care of Animals and followed the Public Health Service guidelines for the humane care and use of experimental animals. Mice were housed in ventilated cages placed in a room maintained at ∼23 °C and 12-h light/dark cycle (lights on from 6:00 a.m. to 6:00 p.m.). The mice had access to tap water and laboratory chow (5L0D; LabDiet) containing 28.5 kcal% protein, 13.5 kcal% fat, and 58.0 kcal% carbohydrate available either ad libitum or restricted according to the approved experimental protocol. Weight-matched Arc*Pomc*^−/−^ (POMC deficiency in the arcuate nucleus) mice were fed 75–80% of the daily total food consumed by their wild type (WT) littermates starting immediately after weaning to prevent their development of obesity. WT and Arc*Pomc*^−/−^ mice were housed individually for experiments involving the calorie restriction. Weight-matched Arc*Pomc*^−/−^ mice were used throughout this study. Generation and breeding of Arc*Pomc*^−/−^ mice, congenic on the C57BL/6J strain, have been described previously [26], [27]. POMC is selectively absent from the ARC, but intact in the nucleus tractus solitarius and the pituitary gland in these mice, due to insertion of a *neomycin* antibiotic selection cassette into the upstream neuronal enhancer locus of the *Pomc* gene.

*Mc4r*^loxP/loxP^ mice [28] were obtained from Dr. David Olson (University of Michigan) with permission from Dr. Bradford Lowell (Harvard University). The *Mc4r*^loxP/loxP^ strain was backcrossed for at least five generations onto the C57BL/6J genetic background before use in this study. Cre recombination-mediated deletion of the *Mc4r* exon sequence flanked by the loxP sites causes a loss of MC4R function.

Non-obese diabetic (NOD) mice and their controls were purchased from the Jackson Laboratory (Stock No: 001976 and 002050, respectively). All of the mice used in this study were male except NOD mice and their controls. Female NOD mice are more susceptible than their male counterparts to developing diabetes. In addition to this polygenic mouse model of type 1 diabetes, we included a pharmacological model of the disease in this study. Type 1 diabetes was chemically induced in mice by administration of streptozotocin (STZ, 50 mg/kg in sodium citrate buffer, pH 5, i.p., Sigma S0130) once daily for five days. Mice that exhibited fasting blood glucose levels >250 mg/dl, two weeks after the first STZ injection, were included in the study.

### 2.2 Insulin induced hypoglycemia and 2-deoxyglucose mediated glucopenia

Mice were fasted for 5-h (9:00 am to 2:00 pm) and following a bolus insulin injection (2 U/kg in PBS, i.p., Humulin R), glycemia was determined at 15, 30, 45, 60, and 120 min. Baseline glycemia was recorded as 0 min measurement. The same procedure was repeated with 2-deoxyglucose (2-DG, 200 mg/kg in PBS, i.p., Sigma D8375) in a separate cohort of mice to induce glucopenia. For the evaluation of hypoglycemia counterregulation in STZ induced diabetes mouse model, the diabetic mice were injected with insulin (10 U/kg, ip) to normalize their basal blood glucose levels 1-h prior to induction of hypoglycemia using the aforementioned protocol. Moreover, additional groups of STZ induced diabetic mice were treated chronically with insulin (10 U/kg/day, ALZET mini-osmotic pump 2002, 14 days) to determine whether the insulin treatment can restore hypothalamic *Pomc* or *Mc4r* expression in addition to normalizing glycemia in diabetes. The cohorts of STZ induced diabetic mice that received either acute or chronic insulin treatments mentioned above are appropriately identified in the figure legends.

### 2.3 Hyperinsulinemic-hypoglycemic clamp

A hyperinsulinemic-hypoglycemic clamp was performed in mice 4 or 5 days after carotid arterial and jugular venous catheterization using the protocol adopted from the Vanderbilt Mouse Metabolic Phenotyping Center [29]. Following a 5-h fast, conscious, freely moving, catheterized mice were infused with insulin (20 mU/kg/min) to clamp glucose at a hypoglycemic level (∼50 mg/dl) for 120 min. The glucose level was maintained via a concomitant glucose infusion at a variable rate. The infusion rate is higher in mice with a defective counterregulatory response to hypoglycemia than those with a normal response because of the inability of the former to restore their blood glucose levels in the face of glucose deficits. Erythrocytes obtained from donor mice via cardiac puncture were infused at 4 μl/min throughout the clamp procedure to prevent a decrease in hematocrit from the repeated blood sampling.

### 2.4 Intracranial surgery and administration of drugs or viral vectors

Mice anesthetized with isoflurane were placed in a stereotaxic frame (Model 1900, Kopf Instruments) and the skull was exposed for intracranial injections or infusions. Coordinates from bregma for administration of drugs or viral vectors were determined using an Allen Brain reference atlas. AAV-Cre-GFP or AAV-GFP (∼50 nl, University of North Carolina Vector Core, titer 8 × 10^12^ vg/ml) were injected bilaterally into the PVH of 6-week old *Mc4r*^loxP/loxP^ mice [coordinates from bregma: anteroposterior, −0.70; mediolateral, ±0.22; dorsoventral, −4.80 mm] using a Picospritzer system (Parker Hannifin) attached to a pulled glass micropipette. Three weeks after the administration of the viral vectors, separate cohorts of *Mc4r*^loxP/loxP + AAV-Cre^ and *Mc4r*^loxP/loxP + AAV-GFP^ mice were subjected to insulin-induced hypoglycemia, 2-DG mediated glucopenia or hyperinsulinemic-hypoglycemic clamps as described above. Weight-matched *Mc4r*^loxP/loxP + AAV-Cre^ mice were used throughout the study.

A subset of STZ treated diabetic mice was infused with melanotan (MT) II bilaterally (1 nmol/day) into the PVH using a brain infusion kit (Plastics1, 3280PD/V/SPC; ALZET mini-osmotic pump 1002). At the 7th day of the MTII infusion, the counterregulatory response to hypoglycemia was measured in separate cohorts of the diabetic mice.

### 2.5 In situ hybridization and qRT-PCR

Fluorescence in situ hybridization (FISH) was performed on 14 μm thick slices of fresh frozen brains using RNAscope^®^ fluorescent multiplex reagents (Advanced Cell Diagnostics, 320850) according to the manufacturer's instructions and a published protocol [30]. RNA probes for *Pomc*, *Mc4r*, and *c-fos* (Advanced Cell Diagnostics, 314081, 402741 and 316921-C2, respectively) were incubated with the brain slices and signal amplification was achieved using the multiplex reagents as described previously [30]. Images were captured using a Nikon 90i upright microscope equipped with an X-Cite 120Q fluorescent light source (Lumen Dynamics) and a CoolSNAP HQ2 CD camera (Photometrics).The RNA signal was quantified by a cell image analysis software (CellProfiler, Broad Institute).

For qRT-PCR, mouse brain was extracted and immediately frozen in 50 ml conical tubes containing isopentane that was pre-chilled on dry ice. The frozen brain was sliced in a cryostat up to −0.4 mm from bregma based on a mouse brain atlas [31] and the PVH (3rd ventricle was used as an anatomical landmark to accurately identify PVH) was punched out bilaterally using a blunt 18G needle. The needle was inserted ∼0.8 mm into the tissue to sample the PVH. For the ARC, we continued the slicing up to −1.46 mm from bregma and the tissue was punched out bilaterally using a separate blunt 18G needle. The needle was inserted ∼0.6 mm into the tissue to sample the ARC. Before punching out the tissues, a 10 μm thick section at the indicated distances from bregma was stained with DAPI and observed under a microscope to validate the accuracy of PVH or ARC microdissections. The punched samples were transferred from the needles into 1.5 ml tubes by applying pressure using 1 ml syringes and stored at −80 °C for the RT-PCR assay. Total RNA from the arcuate or PVH was extracted using RNeasy spin columns (Qiagen) for quantification of *Pomc* or *Mc4r* expression, respectively. After removing genomic DNA with Turbo DNase treatment (Life Technologies), the total RNA was quantified using a NanoDrop spectrophotometer (ThermoScientific). Reverse transcription to generate cDNA was performed with 500 ng total RNA and random hexamer primers (Goscript RT System, Promega). qRT-PCR was performed on all samples in duplicate using a StepOne Real Time PCR System (Applied Biosystems) and SYBR Green Master Mix (Life Technologies). The primers for detection of *Pomc* were: 5′-GAGCTGGTGCCTGGAGAG-3′ and 5′-TTTTCATCAGGGGCTGTTC-3′ (designed to span exons 2 and 3 of splice variant 1); for *Mc4r*, 5′-GGAAGATGAACTCCACCCACC-3′ and 5′-AATGGGTCGGAAACCATCGTC-3′. All primers were used at a final concentration of 300 nM. The relative quantity of each mRNA was calculated from standard curves spanning 1000-fold change, normalized to a reference gene *Hprt* and then normalized to the mean of control.

### 2.6 Epinephrine and glucagon assays

Plasma epinephrine and glucagon were measured at the indicated time points using ELISAs according to manufacturers' instructions (epinephrine, BA E-5100, LDN; glucagon, 10-1281-01, Mercodia).

### 2.7 Statistical analyses

Two-tailed Student's unpaired t-test was used to compare one dependent variable between two independent groups. One-way ANOVA followed by Tukey's multiple comparison test was applied for comparisons among three or more independent groups for one dependent variable. Comparisons between two independent groups involving one dependent variable with repeated measures were made by repeated measures two-way ANOVA (RMANOVA) followed by Tukey's multiple comparison test. All analyses were performed using Prism 7.0 (Graph Pad) and P < 0.05 was considered significant.

## 3 Results

### 3.1 Hypoglycemia as well as glucopenia activates POMC and MC4R neurons

**Figure 1:**
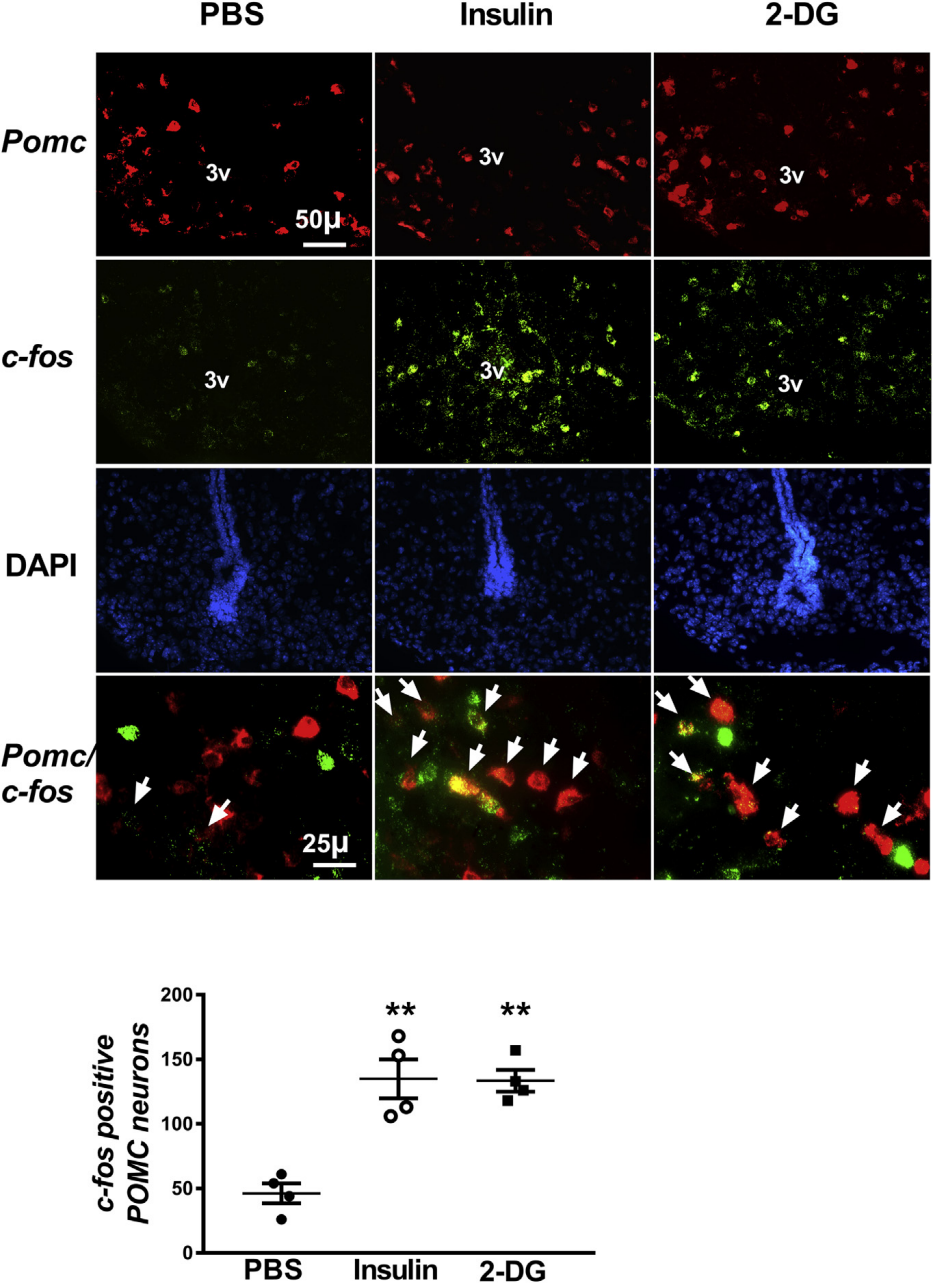
Activation of POMC neurons in the arcuate nucleus by hypoglycemia or glucopenia in 8-week old male WT mice. Representative images of fluorescence in situ hybridization following insulin-induced hypoglycemia or 2-deoxyglucose (2-DG) mediated glucopenia. Higher number of *c-fos* positive POMC neurons in the arcuate nucleus 30 min after insulin (2 U/kg, ip) or 2-DG (200 mg/kg, ip) treatment compared to PBS administration. Arrowheads point to colocalization of *Pomc* (red) and *c-fos* (green) imaged using a 40× objective lens. POMC neuron profiles overlapping with at least 3 green dots (representing *c-fos*) per neuron were included in the analysis for counting the number of *c-fos* positive POMC neurons. All other images were captured under a 20× objective lens. Quantification of fluorescence signal was performed using CellProfiler. **P < 0.01 for insulin or 2-DG vs PBS (n = 4, 4 sections per mouse and 4 areas of interest per section were analyzed), 1-way ANOVA followed by Tukey's multiple comparisons test. Error bars reflect mean ± SEM.

**Figure 2:**
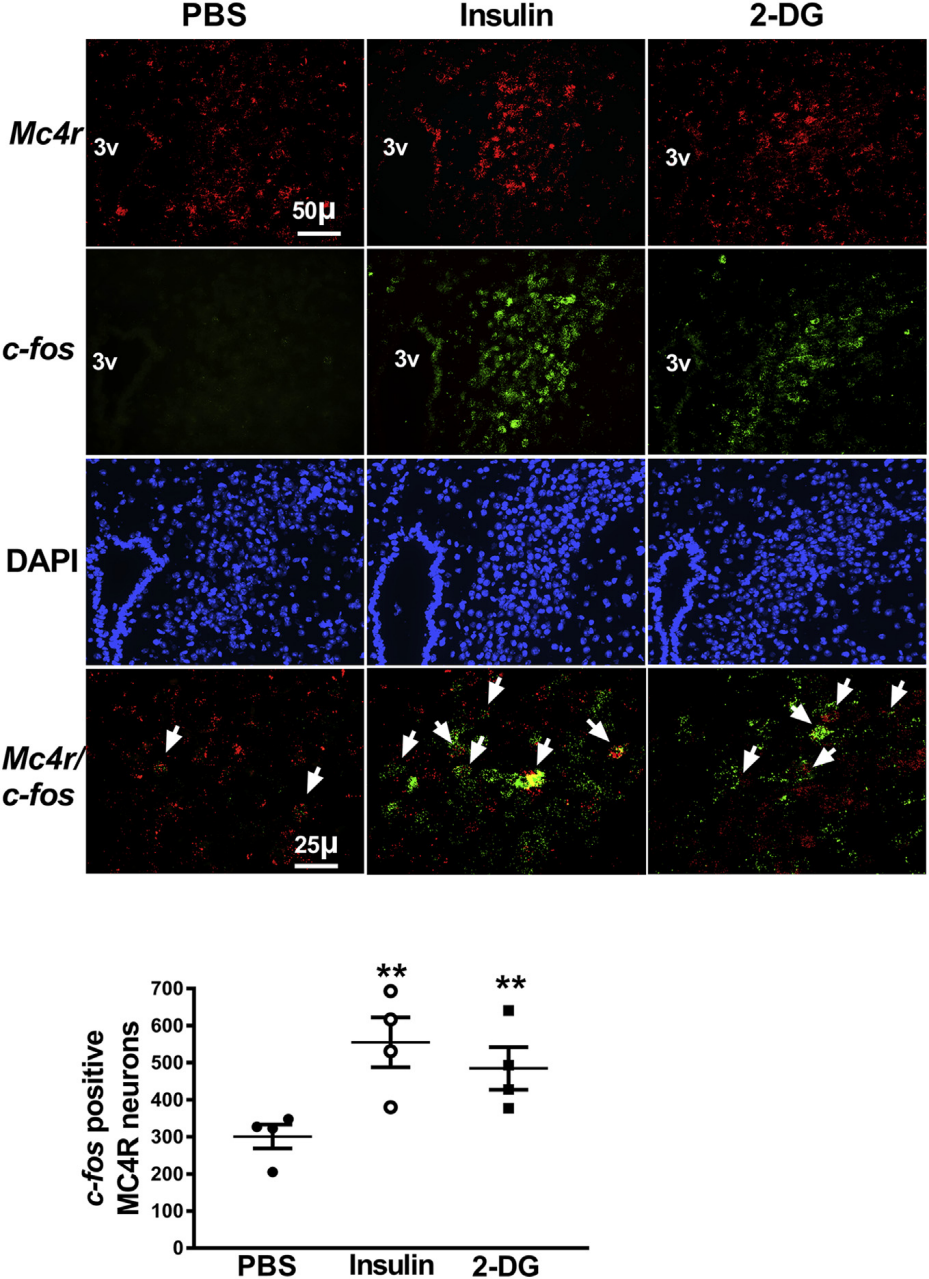
Activation of MC4R neurons in the paraventricular nucleus of the hypothalamus by hypoglycemia or glucopenia in 8-week old male WT mice. Representative images of fluorescence in situ hybridization following insulin-induced hypoglycemia or 2-deoxyglucose (2-DG) mediated glucopenia. Increase in number of *c-fos* positive MC4R neurons in the paraventricular nucleus 30 min after insulin (2 U/kg, ip) or 2-DG (200 mg/kg, ip) treatment compared to PBS administration. Arrowheads point to colocalization of *Mc4r* (red) and *c-fos* (green) imaged using a 40× objective lens. MC4R neuron profiles overlapping with at least 3 green dots (representing *c-fos*) per neuron were included in the analysis for counting the number of *c-fos* positive MC4R neurons. All other images were captured with a 20× objective lens. Quantification of fluorescence signal was performed using CellProfiler. **P < 0.01 for insulin or 2-DG vs PBS (n = 4, 4 sections per mouse and 4 areas of interest per section were analyzed), 1-way ANOVA followed by Tukey's multiple comparisons test. Error bars reflect mean ± SEM.

We employed FISH using RNAScope reagents [30] to determine if hypoglycemia or glucopenia activate POMC and MC4R neurons in ARC and PVH, respectively. Separate cohorts of WT mice were fasted for 5-h before administration of PBS or insulin (2 U/kg, ip) or 2-DG (200 mg/kg, ip). The mice were decapitated and their brains removed at 30 min after the injections. Fourteen μm thick sections were probed for the expression of *Pomc* or *Mc4r* along with *c-fos*, a neuronal activation marker. The expression of *c-fos* was significantly higher in POMC (Figure 1) and MC4R neurons (Figure 2) in mice after insulin or 2-DG treatment compared to those injected with PBS, indicating that the neurons were activated following hypoglycemia or glucopenia. The neurons labeled with at least three green dots per neuron were considered *c-fos* positive for quantitative analyses. The total number of DAPI-labeled neurons was unchanged among the groups (POMC: PBS – 467 ± 56, Insulin – 430 ± 105, 2-DG – 503 ± 63; MC4R: PBS – 935 ± 32, Insulin – 870 ± 77, 2-DG – 913 ± 53, mean ± SEM). It is important to note that not all of the POMC or MC4R neurons were activated following hypoglycemia or glucopenia probably because of the heterogeneity of these neurons, as already reported for POMC neurons [32]; nevertheless, the number of activated neurons was higher in the insulin or 2-DG treated groups compared to the control mice.

### 3.2 Hypothalamic POMC and MC4R are indispensable for hypoglycemia counterregulation

**Figure 3:**
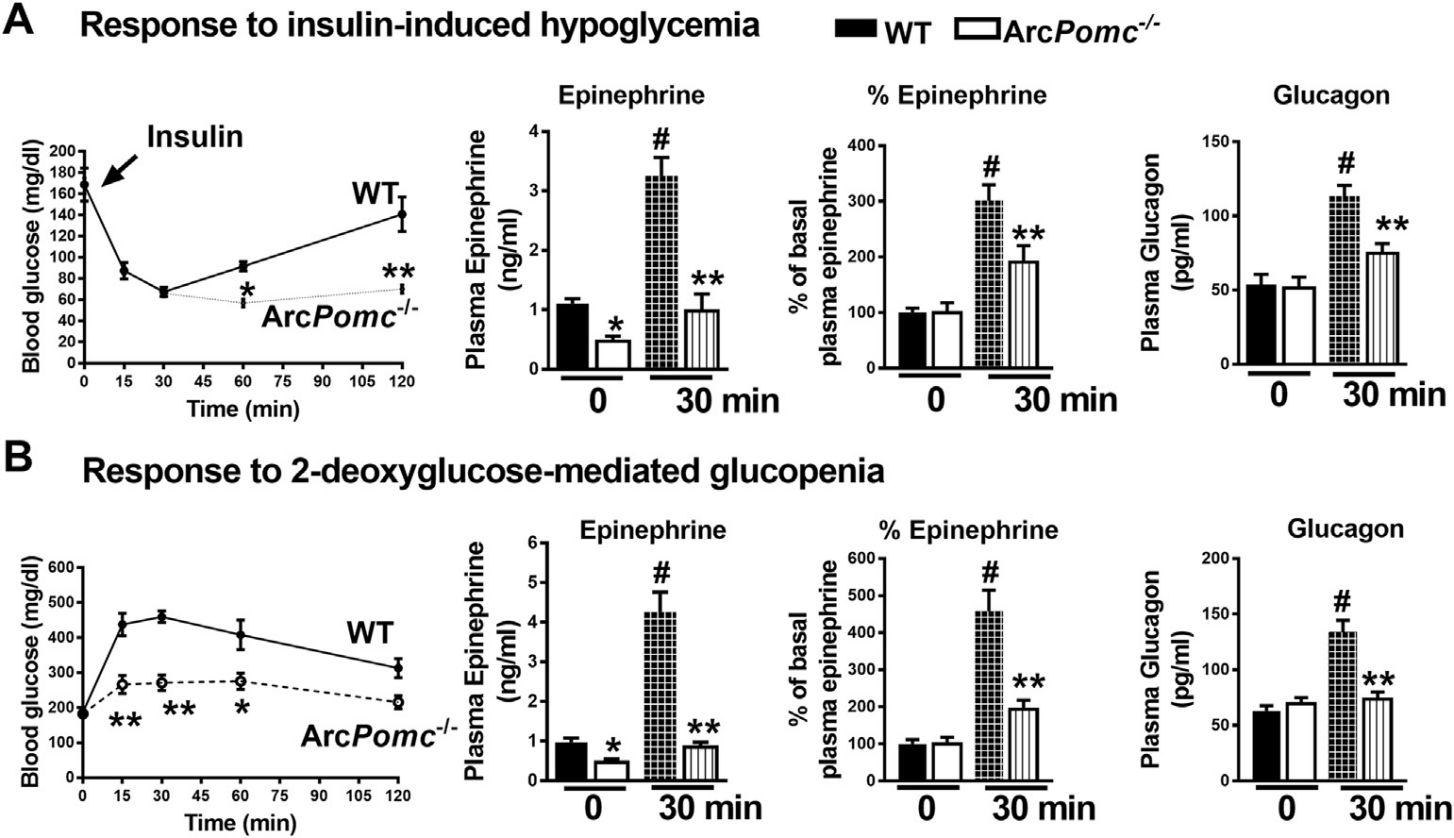
*Pomc* in the arcuate nucleus is essential to counteract hypoglycemia or glucopenia. **A)** Restoration of baseline glycemia is impaired together with reduced stimulation of release of epinephrine (absolute values and % change) and glucagon in Arc*Pomc*^−/−^ mice following insulin-induced hypoglycemia; **B)** Diminished response to glucose deficit, impaired stimulation of release of epinephrine (absolute values and % change) and glucagon in Arc*Pomc*^−/−^ mice following 2-deoxyglucose (2-DG) mediated glucopenia. 2-way RMANOVA followed by Tukey's multiple comparison test was used for comparisons. For the blood glucose levels, *P < 0.05, **P < 0.01 vs WT; for the epinephrine and glucagon values, *P < 0.05 vs WT at 0 min, **P < 0.01 vs WT at 30 min, ^#^P < 0.01 vs both the groups at 0 min and Arc*Pomc*^−/−^ at 30 min, n = 6–8. Error bars are mean ± SEM.

We subjected WT and weight-matched Arc*Pomc*^−/−^ mice to hypoglycemia induced by a high dose of insulin (2 U/kg, ip). This dose was sufficient to cause hypoglycemia in otherwise insulin-resistant Arc*Pomc*^−/−^ mice [27]. Interestingly, unlike in WT mice, normal blood glucose levels were not restored in Arc*Pomc*^−/−^ mice following hypoglycemia (Figure 3A). Baseline plasma epinephrine, but not glucagon, levels were lower in Arc*Pomc*^−/−^ mice compared to their WT littermates (Figure 3A). Furthermore, release of both of the counterregulatory hormones at 30 min was blunted in Arc*Pomc*^−/−^ mice (Figure 3A), indicating the contribution of hypothalamic POMC in counteracting hypoglycemia. The basal plasma epinephrine was significantly lower in the Arc*Pomc*^−/−^ mice, and there was only a twofold increase in plasma epinephrine in these mice compared to a threefold increase in the WT mice in response to hypoglycemia. These data demonstrate that the Arc*Pomc*^−/−^ mice had suppressed release of epinephrine following hypoglycemia independently of basal plasma epinephrine levels.

To test if hypothalamic POMC is involved in counteracting glucopenia independently of insulin actions, we evaluated the counterregulatory response in 2-DG treated Arc*Pomc*^−/−^ mice. Consistent with the data from the insulin-induced hypoglycemia model, the Arc*Pomc*^−/−^ mice had impaired responses to 2-DG-induced glucopenia (Figure 3B). Increased glucose clearance by glycosuria [27] may have impacted glycemia at different time points, however, plasma epinephrine and glucagon levels were clearly lower in the Arc*Pomc*^−/−^ mice relative to the WT mice in response to 2-DG-induced glucopenia (Figure 3B). Hence, these findings indicate the contribution of hypothalamic POMC in defending against glucose deficit independently of its underlying cause.

**Figure 4:**
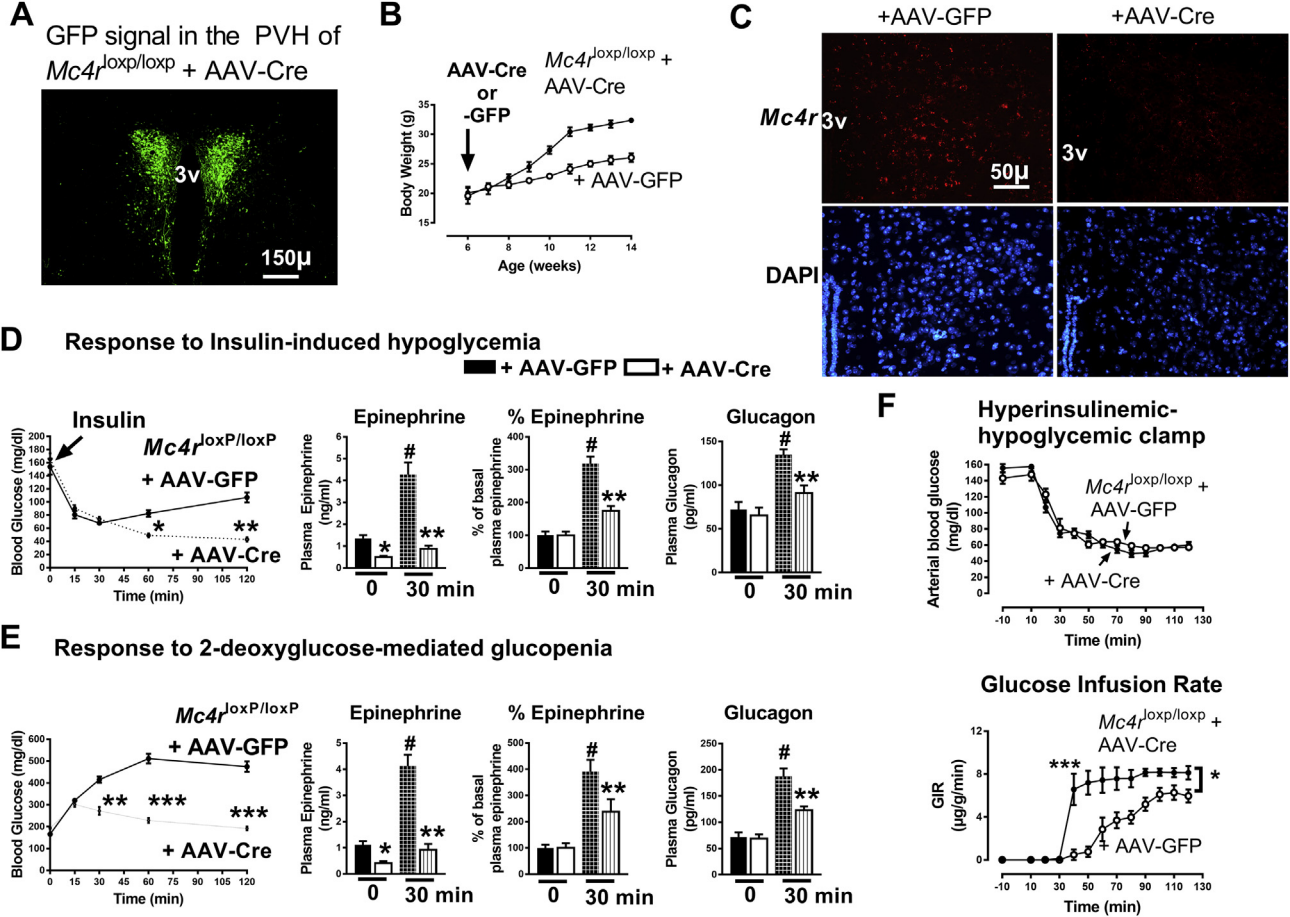
*Mc4r* in the paraventricular nucleus is required to counteract glucose deficit. **A)** Representative image of GFP signal confined to the paraventricular nucleus of the hypothalamus (PVH), validating the accuracy of AAV-Cre-GFP injections in *Mc4r*^loxP/loxP^ mice; **B)** Increased body weight in *Mc4r*^loxP/loxP + AAV-Cre^ mice; **C)** Representative images of fluorescence in situ hybridization showing decreased *Mc4r* levels in *Mc4r*^loxP/loxP + AAV-Cre^ mice.; **D)** Restoration of baseline glycemia is impaired together with reduced stimulation of release of epinephrine (absolute values and % change) and glucagon in 9-week old *Mc4r*^loxP/loxP^ mice injected with AAV-Cre (generating PVH-specific *Mc4r* deficiency) following insulin-induced hypoglycemia; **E)** Diminished response to glucose deficit, impaired stimulation of release of epinephrine (absolute values and % change), and glucagon in 9-week old *Mc4r*^loxP/loxP^ mice injected with AAV-Cre (generating PVH-specific *Mc4r* deficiency) following 2-deoxyglucose (2-DG) mediated glucopenia; **F)** Glucose infusion rate (GIR) in 12-week old male *Mc4r*^loxP/loxP + AAV-Cre^ and *Mc4r*^loxP/loxP + AAV-GFP^ mice during hyperinsulinemic-hypoglycemic clamps, glucose clamped at hypoglycemic level using 20 mU/kg/min insulin. 2-way RMANOVA followed by Tukey's multiple comparison test was used for comparisons. For the blood glucose levels, *P < 0.05, **P < 0.01, ***P < 0.001 vs *Mc4r*^loxP/loxP + AAV-GFP^; for the epinephrine and glucagon values, *P < 0.05 vs *Mc4r*^loxP/loxP + AAV-GFP^ at 0 min, **P < 0.01 vs *Mc4r*^loxP/loxP + AAV-GFP^ at 30 min, ^#^P < 0.01 vs both the groups at 0 min and *Mc4r*^loxP/loxP + AAV-Cre^ at 30 min; for the GIR, *P < 0.05, ***P < 0.001 vs *Mc4r*^loxP/loxP + AAV-GFP^, n = 6–8. Error bars are mean ± SEM.

MC4R in the PVH (MC4R^PVH^) mediates the actions of POMC peptides on energy balance [33], [34]. Therefore, to determine the mechanisms underlying POMC control of hypoglycemia counterregulation, we studied the contribution of MC4R^PVH^ in stimulating the release of epinephrine and glucagon. We injected AAV-Cre bilaterally into the PVH of *Mc4r*^loxP/loxP^ mice to knockdown *Mc4r* specifically in the PVH. The accuracy of the AAV-Cre administration in the PVH of Mc4r^loxP/loxP + AAV-Cre^ mice was determined at the end of the study by confirming a GFP signal in the PVH using a fluorescence microscope. Data from only those *Mc4r*^loxP/loxP + AAV-Cre^ mice that accurately showed the signal confined to the PVH (Figure 4A) were included in the analyses. Moreover, the accuracy of viral vector targeting in the *Mc4r*^loxP/loxP^ mouse model was further validated by an increase in body weight in *Mc4r*^loxP/loxP + AAV-Cre^ mice compared to *Mc4r*^loxP/loxP + AAV-GFP^ mice (Figure 4B) as reported previously [28]. Deficiency of *Mc4r* in *Mc4r*^loxP/loxP + AAV-Cre^ mice was confirmed by measuring its expression in the PVH using FISH (Figure 4C). Quantitative analyses of the FISH data showed that the number of cells expressing *Mc4r* was significantly lower in *Mc4r*^loxP/loxP + AAV-Cre^ mice than that observed in *Mc4r*^loxP/loxP + AAV-GFP^ mice (AAV-GFP: 1286 ± 64 vs. AAV-Cre: 316.5 ± 49, n = 4, 4 sections per mouse and 4 areas of interest per section were analyzed, P < 0.001), thereby further validating the deficiency of *Mc4r* in the PVH in the former.

Separate cohorts of the PVH-specific MC4R-deficient mice (*Mc4r*^loxP/loxP + AAV-Cre^) were subjected to insulin-induced hypoglycemia or 2-DG-induced glucopenia using the same protocol as described for Arc*Pomc*^−/−^ mice. In agreement with the results obtained from the Arc*Pomc*^−/−^ mice, *Mc4r*^loxP/loxP + AAV-Cre^ mice exhibited a reduced counterregulatory response to hypoglycemia (Figure 4D) or glucopenia (Figure 4E) compared to *Mc4r*^loxP/loxP + AAV-GFP^ mice. Like the Arc*Pomc*^−/−^ mice, the PVH-specific MC4R-deficient mice had lower basal plasma epinephrine levels relative to their littermate controls. Moreover, the MC4R-deficient mice exhibited less than a twofold increase compared to a threefold increase in plasma epinephrine levels in the controls in response to hypoglycemia. These data indicate that the PVH-specific MC4R-deficient mice had reduced release of epinephrine following hypoglycemia independently of basal plasma epinephrine levels. We also performed hyperinsulinemic-hypoglycemic clamps in *Mc4r*^loxP/loxP + AAV-Cre^ mice to further assess their response to hypoglycemia. The glucose infusion rate (GIR) was higher in *Mc4r*^loxP/loxP + AAV-Cre^ mice compared to *Mc4r*^loxP/loxP + AAV-GFP^ mice (Figure 4F), indicating an impaired ability in the former to counter hypoglycemia. There was no difference in plasma insulin levels between the groups at the end of the clamp experiment at 120 min (*Mc4r*^loxP/loxP + AAV-Cre^: 33.73 ± 8 vs. *Mc4r*^loxP/loxP + AAV-GFP^: 32.11 ± 8.5 ng/ml, n = 7).

### 3.3 Reduced hypothalamic *Pomc* and *Mc4r* expression in type 1 diabetes mouse models

**Figure 5:**
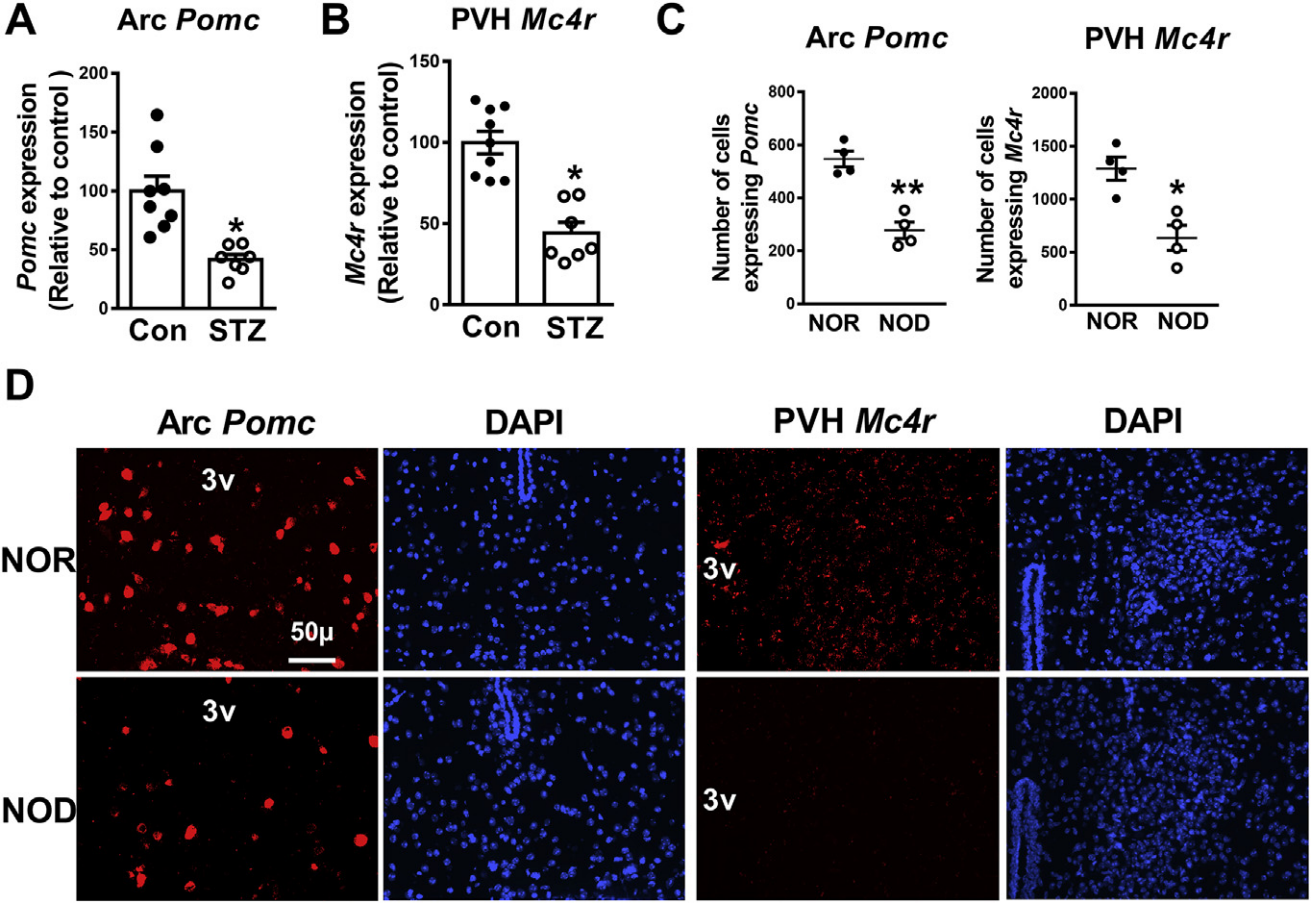
Reduced expression of hypothalamic *Pomc* and *Mc4r* in type 1 diabetes mouse models. **A)** Data from qRT-PCR showing decreased *Pomc* mRNA levels in the arcuate nucleus (Arc) in streptozotocin (STZ) induced diabetes mouse model and control (con) group (12-week old mice); **B)** Data from qRT-PCR showing reduced *Mc4r* in the paraventricular nucleus (PVH) in STZ induced diabetes mouse model and control group (12-week old mice); **C)** Quantification of fluorescence in situ hybridization signal for *Pomc* and *Mc4r* in 30-week old female NOR (non-obese diabetes resistant) and NOD (non-obese diabetic) mice for the representative images shown in **D** (n = 4, 4 sections per mouse and 4 areas of interest per section were analyzed). 2-tailed Student's t-test was used for comparisons. *P < 0.05, **P < 0.01 vs control or NOR group. Error bars are mean ± SEM. control, mice injected with Na-Citrate Buffer.

The counterregulatory response to hypoglycemia is impaired in diabetes. To elucidate potential molecular mechanisms underlying the impairment, we determined the expression of *Pomc* and *Mc4r* in the ARC and PVH, respectively, in STZ treated diabetic and NOD mice. qRT-PCR was used to measure the mRNA levels in the STZ treated mice and FISH was employed for the NOD mice. The expression of both *Pomc* and *Mc4r* was significantly reduced (Figure 5A,B) four weeks after the last injection of STZ. Female NOD mice (30-week old) also exhibited a decrease in the expression of *Pomc* and *Mc4r* (Figure 5C,D).

**Figure 6:**
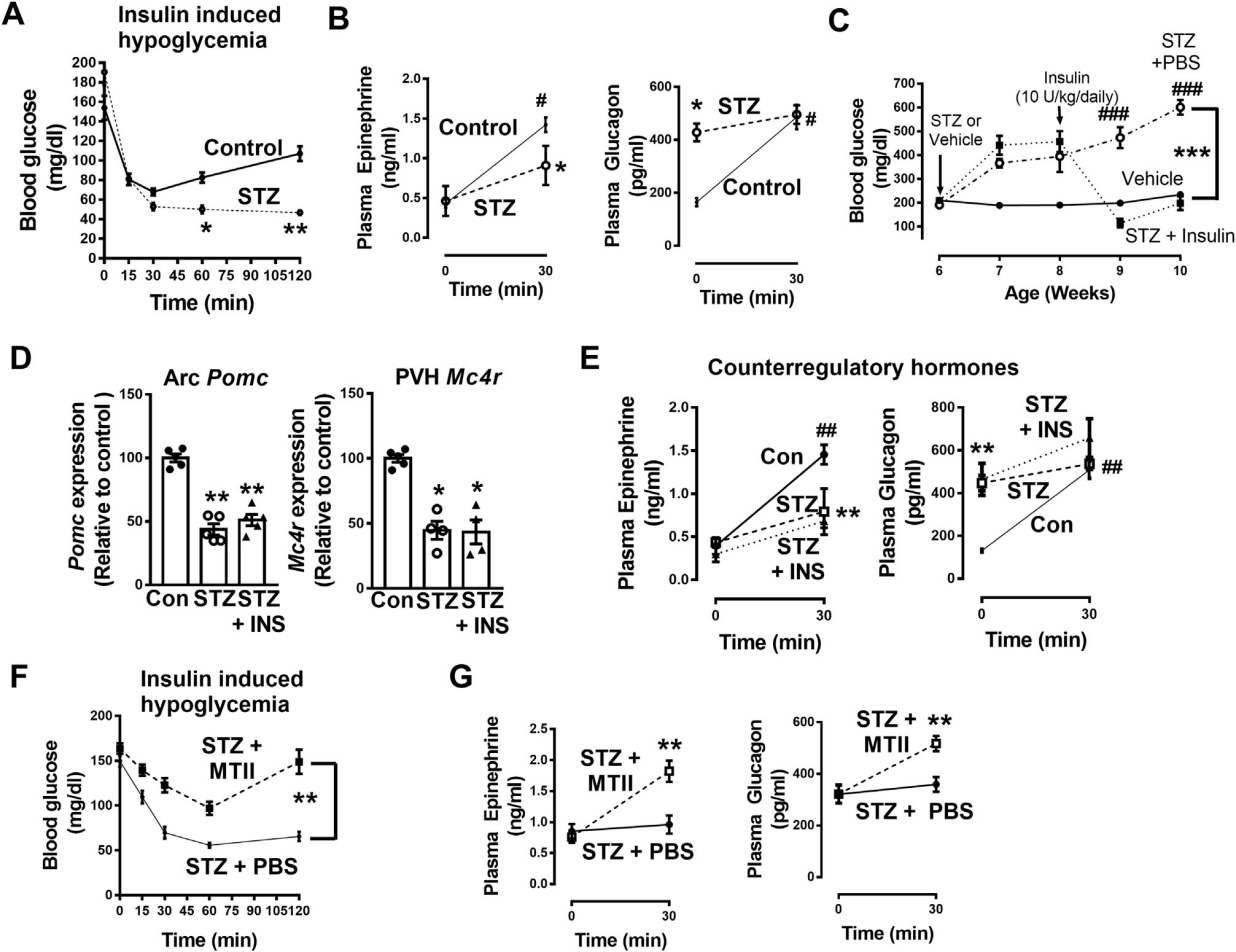
Hypoglycemia counterregulatory response and effects of chronic insulin or MC4R agonist infusion in the streptozotocin (STZ) induced diabetes mouse model. **A)** Restoration of baseline glycemia is impaired in 12-week old STZ treated mice following insulin-induced hypoglycemia (STZ mice were pretreated with 10 U/kg insulin, ip, 1-h prior to this experiment to match their baseline glycemia with control group), *P < 0.05, **P < 0.01 vs control; **B)** Reduced stimulation of epinephrine and glucagon release in the STZ treated mice following insulin-induced hypoglycemia, *P < 0.05 vs control at 30 min for epinephrine and at 0 min for glucagon, ^#^P < 0.05 vs control at 0 min**; C)** Restoration of normoglycemia in diabetic mice by insulin treatment (10 U/kg/day, 14 days), ***P < 0.001 vs vehicle, ^###^P < 0.001 vs STZ + Insulin, n = 6; **D)** Insulin (INS) treatment did not normalize either *Pomc* or *Mc4r* mRNA levels in diabetic mice, *P < 0.05, **P < 0.01 vs control, n = 5; **E)** Insulin treatment did not enhance the release of counterregulatory hormones in diabetic mice, **P < 0.01 vs control at 30 min for epinephrine and at 0 min for glucagon, ^##^P < 0.01 vs control at 0 min, n = 6; **F)** Melanotan (MT) II (1 nmol/day, PVH) improves counterregulatory response to hypoglycemia in diabetes induced by STZ (the mice were pretreated with 10 U/kg insulin, ip, 1-h prior to this experiment to normalize their baseline glycemia), **P < 0.01 vs STZ, n = 6; **G)** MT II increased the release of counterregulatory hormones in the diabetic mice,**P < 0.01 vs STZ. 1-way ANOVA or 2-way RMANOVA followed by Tukey's multiple comparison test were used for comparisons as appropriate. Error bars are mean ± SEM. Vehicle or control, Na-Citrate Buffer.

In addition to measuring the *Pomc* and *Mc4r* expression, we evaluated hypoglycemia counterregulatory response in the STZ diabetic mice. As expected, the diabetic mice exhibited reduced counterregulatory response to hypoglycemia (Figure 6A,B). It is important to note that, in accordance with previous observations [35], [36], basal glucagon levels were elevated in the diabetic mice. Moreover, there was no increase in the plasma glucagon levels in response to hypoglycemia at 30 min in the diabetic mice. The STZ-induced diabetic mice were injected with insulin (10 U/kg, ip) to match their basal glycemia with that of non-diabetic control 1-h prior to being evaluated for their hypoglycemia counterregulatory response. Collectively, these data indicate that the impairment of the counterregulatory responses in diabetes may be associated with the reduced hypothalamic *Pomc* and *Mc4r* expression.

### 3.4 Chronic insulin treatment did not restore *Pomc* or *Mc4r* expression despite reversing hyperglycemia

Insulin corrects hyperglycemia; however, it is unclear if it reverses other abnormalities caused by diabetes. We determined the impact of chronic insulin subcutaneous infusion (10 U/kg/day, ALZET mini-osmotic pump 2002, 14 days) on *Pomc* or *Mc4r* expression in STZ treated diabetic mice. While the insulin therapy corrected hyperglycemia (Figure 6C) in the diabetic mice, it did not restore *Pomc* or *Mc4r* expression in ARC or PVH (Figure 6D), respectively. Moreover, the treatment did not enhance the release of counterregulatory hormones (Figure 6E). These data indicate the need for an additional intervention that either restores *Pomc* and *Mc4r* expression levels or acts downstream of the hypothalamic melanocortin circuit to correct the defective hypoglycemia counterregulation in diabetes.

### 3.5 MC4R agonist restored counterregulatory response to hypoglycemia in diabetes

MTII is an MC4R agonist. Given the reduced *Mc4r* expression in the STZ treated mice (Figure 5B), we hypothesized that enhancing MC4R signaling via MTII administration may improve the counterregulatory response to insulin induced hypoglycemia in diabetes. To test this hypothesis, we infused PBS or MTII (1 nmol/day, ALZET mini-osmotic pump 1002) into the PVH bilaterally for 7 days in mice with diabetes induced by STZ. Remarkably, the MTII treatment improved counterregulatory responses to hypoglycemia (Figure 6F) in the diabetic mice by augmenting the stimulation of epinephrine and glucagon release (Figure 6G) compared to the diabetic mice that received PBS. It is important to note that at 30 min following insulin injection, the MTII treated diabetic mice did not exhibit hypoglycemia (∼120 mg/dl, Figure 6F). Despite this absence of hypoglycemia, plasma epinephrine and glucagon levels at that time were higher in the MTII treated diabetic mice than that in the PBS-infused diabetic mice. These data indicate that MTII treatment might help defend against even a moderate decrease in blood glucose levels from the baseline in the presence of insulin by stimulating the release of counterregulatory hormones. While we did not measure epinephrine and glucagon levels at 60 min post insulin injection, it is very likely that the levels were higher than what we observed at 30 min. Overall, these data demonstrate the feasibility of using an MC4R agonist to defend against hypoglycemia in diabetes.

## 4 Discussion

The primary goal of this study was to determine the function of POMC and MC4R, which is a receptor for POMC derived melanocyte-stimulating hormones, in hypoglycemia counterregulation. We report that POMC in the ARC and MC4R in the PVH are indispensable to counteract hypoglycemia or glucopenia. Moreover, we have demonstrated reduced expression of *Pomc* and *Mc4r* in the ARC and PVH, respectively, in two different mouse models of type 1 diabetes. Insulin treatment did not restore the expression of these genes. Remarkably, administration of an MC4R agonist improved hypoglycemia counterregulation in diabetic mice.

The VMH is a major glucose-sensing center that is essential in orchestration of the counterregulatory response to hypoglycemia [11], [12], [13]. Moreover, the hindbrain is necessary for mobilizing energy through behavioral and neuroendocrine responses to overcome a glucose deficit [37]. Neurons in the ARC and PVH are among the first responders to defend against hypoglycemia [38], [39], [40]. Despite these advances in our understanding of the type of neurons involved in hypoglycemia counterregulation, molecular mechanisms responsible for such coordinated defenses against hypoglycemia remain unclear. Given the contribution of the hypothalamic POMC/MC4R circuit in regulating the SNS [17], [18], [19], [20], [21], [22], [23], [24], we determined the function of POMC and MC4R in hypoglycemia counterregulation.

Using FISH to detect cfos mRNA, we demonstrated that POMC and MC4R neurons in the ARC and PVH, respectively, were activated following hypoglycemia. Moreover, we employed Arc*Pomc*^−/−^ mice and *Mc4r*^*PVH*^-knockdown mice to understand the contribution of POMC and MC4R in hypoglycemia counterregulation. Both of these mouse models exhibited reduced counterregulatory responses to hypoglycemia and glucopenia. It is known that global POMC deficiency also leads to impaired hypoglycemia counterregulation in mice [41]. Moreover, humans with POMC mutations have been reported to exhibit hypoglycemia [42]. In both cases, the global loss of *Pomc* expression leads to the absence of circulating adrenal glucocorticoids that are required for maintenance of normal glycemia. Arc*Pomc*^−/−^ mice on the other hand have normal *Pomc* expression in pituitary corticotrophs [26] thus preventing hypocorticosteronemia. In the context of these findings, we have demonstrated the significance of hypothalamic POMC alone in hypoglycemia counterregulation including stimulation of the release of epinephrine and glucagon. We report that responses to insulin induced hypoglycemia and 2-DG mediated glucopenia are significantly impaired in the Arc*Pomc*^−/−^ mice. The Arc*Pomc*^−/−^ mice exhibit insulin resistance with a low insulin dose of 0.75 U/kg [27]. However, when insulin was administered at a dose of 2 U/kg in this current study, the Arc*Pomc*^−/−^ mice failed to restore their glycemia due to reduced release of epinephrine and glucagon.

MC4R is one of the most predominant receptors downstream of POMC peptides in the hypothalamus [33], [34] and the hypothalamic POMC/MC4R circuit is essential for energy balance [34], [43], [44], [45]. However, the contribution of MC4R^PVH^ in hypoglycemia counterregulation remains undefined. MC4R-deficient mice and humans with MC4R mutations exhibit reduced sympathetic nervous system activity [17], [18], [19], [20], [21], [22], [23]. Therefore, MC4R^PVH^ appeared to be a likely candidate, downstream of ArcPOMC, in mediating the counterregulatory response to hypoglycemia. Like the Arc*Pomc*^−/−^ mice, the *Mc4r*^*PVH*^-knockdown mice manifested reduced circulating epinephrine and glucagon following hypoglycemia. These data corroborate a previous report that MC4R activation is involved in stimulating sympathetic preganglionic neurons [46], which, in turn, contribute toward release of the counterregulatory hormones [47], [48], [49]. In addition to the high-dose insulin induced hypoglycemia and 2-DG mediated glucopenia, we employed the hyperinsulinemic-hypoglycemic clamp technique to further validate the phenotype of an impaired hypoglycemia counterregulation in the *Mc4r*^*PVH*^-knockdown mice. The *Mc4r*^*PVH*^-knockdown mice exhibited higher GIR, indicating an impaired endogenous ability to counteract hypoglycemia. Overall, these findings demonstrate that MC4R in the PVH is essential for hypoglycemia counterregulation.

Both the Arc*Pomc*^−/−^ and *Mc4r*^*PVH*^-knockdown mice had reduced basal plasma epinephrine but not glucagon levels. This phenotype is likely due to differential regulation of epinephrine-secreting chromaffin cells and glucagon-releasing α-cells by the hypothalamic POMC/MC4R circuit. For example, it was recently reported that MC4Rs in the lateral hypothalamus differentially regulate glucose tolerance independently of energy balance [22]. In our current study, we did not determine the downstream pathways underlying the discrepancy between the basal plasma epinephrine and glucagon levels, which is a limitation of this report. Moreover, we did not ascertain whether the defective glucagon release in response to hypoglycemia or glucopenia in the Arc*Pomc*^−/−^ and *Mc4r*^*PVH*^-knockdown mice was secondary to the reduced basal epinephrine levels. Indeed, it is known that an increase in plasma glucagon in response to glucose deficits may be attributed, at least in part, to elevated circulating epinephrine levels [50]. Therefore, further experiments are required to delineate the underlying downstream pathways through which hypothalamic POMC or MC4R differentially regulates epinephrine and glucagon release.

Hypoglycemia counterregulation is impaired in diabetes [2], [3], [4] probably due to sympathetic neuropathy [47], [51], [52]. Therefore, life-threatening hypoglycemia is a major limiting factor in the treatment of diabetes. To understand molecular mechanisms elucidating the impaired hypoglycemia, we determined the expression of *Pomc* and *Mc4r* in the ARC and PVH, respectively, in type 1 diabetes mouse models. Steady state levels of mRNA for both genes were reduced in STZ induced diabetic and NOD mice, demonstrating the weakening of the system that is required for hypoglycemia counterregulation. Our results concur with previous reports [53], [54] indicating that diabetes reduces hypothalamic melanocortin signaling. Moreover, the reported findings [54] further support our data that insulin treatment does not completely restore *Pomc* expression in the ARC during diabetes. In contrast, it was also reported that 21-day insulin treatment restores *Pomc* expression in the ARC in diabetic rats [55]. Nevertheless, *Mc4r* expression was not measured in those previous studies; our current findings demonstrate that insulin treatment does not restore *Mc4r* expression in the PVH. These data warrant the need for an intervention to restore hypothalamic melanocortin signaling and correct the impaired counterregulatory response to hypoglycemia in diabetes. To achieve this goal, we assessed the ability of MTII in restoring a normal counterregulatory response in the STZ induced diabetic mice. Indeed, the MTII treatment increased the release of epinephrine and glucagon in response to even a moderate decrease in blood glucose levels from the baseline. Setmelanotide, an MC4R agonist, is already undergoing a clinical trial for the treatment of obesity [55]. Based on the preclinical results of our study, setmelanotide could also be useful to correct sympathetic neuropathy and improve counterregulatory responses to hypoglycemia in diabetes.

In conclusion, hypothalamic *Pomc* as well as *Mc4r*, both of which are reduced in type 1 diabetic mice, are required for normal counterregulatory responses to hypoglycemia. Therefore, enhancing MC4R function may minimize the risk of life-threatening hypoglycemia caused by insulin or its secretagogues during the treatment of diabetes.
